# Lightweight super-resolution reconstruction architecture of remote sensing images using a residual hierarchical transformer network

**DOI:** 10.1038/s41598-025-28042-1

**Published:** 2025-12-23

**Authors:** Bo Huang, Jian Lin, Qingtang Chen, Yiqing Cao, Liaoni Wu

**Affiliations:** 1 College of Intelligent Manufacturing, Putian University, Putian, 351100 China; 2 Fujian Laser Precision Machining Engineering Technology Research Center, Putian, 351100 China; 3 School of Aerospace Engineering, Xiamen University, Xiamen, 361102 China

**Keywords:** Engineering, Mathematics and computing

## Abstract

Remote Sensing image super-resolution technology aims to enhance spatial details, and it is of great significance for the high-quality interpretation of satellite imagery. Recently, Transformer-based models have shown competitive performance in single image super-resolution (SISR). However, current Transformer-based SR approaches often employ window self-attention with fixed small window sizes, limiting the receptive filed to a single scale and preventing the network from gathering multi-scale information such as local textures and repetitive patterns, impeding the model’s ability to remote sensing images. Moreover, the quadratic computational complexity resulting from global self-attention, rendering it inefficient for addressing remote sensing SISR tasks that involve processing high-resolution images. To address these issues, we proposed a vision transformer architecture called residual hierarchical transformer network (RHTN). Specifically, we have developed a residual hierarchical transformer block (RHTB) as a building block in RHTN. In the RHTB, we introduce a novel spatial-channel self-attention mechanism characterized by linear complexity relative to window dimensions. This design optimally harvests both spatial structural information and channel-wise features from the hierarchical window framework while maintaining computational tractability. Then, we adopt the spatial-gate feed-forward network to further model additional non-linear spatial information. We conducted comprehensive experiments on multiple benchmark datasets, demonstrating the superior performance of our proposed RHTN in terms of quantitative metrics and visual quality when compared to state-of-the-art methods.

## Introduction

The rapid advancement of remote sensing satellite communication and unmanned aerial vehicle (UAV) technology has significantly expanded the application potential of remote sensing imagery in critical domains, including marine observation^1,2^, disaster monitoring^3,4^, urban planning^5–7^, and natural resource exploration^8,9^. While these systems have brought new opportunities for earth observation technology, they simultaneously introduce new challenges to remote sensing image processing. Due to atmospheric interference, long-distance imaging conditions, and limitations in camera channel transmission efficiency, acquired images frequently suffer from degraded spatial resolution, thereby restricting their utility in high-precision applications. Given the substantial research costs and prolonged hardware iteration cycles associated with physically enhancing imaging sensors, there is a growing imperative to optimize algorithmic approaches for super-resolution (SR) reconstruction^10^ of remote sensing images.

With the flourishing development of deep learning and big-data technology, promising results have been obtained in computer vision tasks^11,12^. Since the groundbreaking work of SRCNN^13^ that first introduced the convolutional neural network (CNN) into the SR reconstruction field, various CNN-based methods have emerged^14–17^. Although CNN-based methods have brought significant progress to the development of SR tasks, the limitations of convolutional kernels prevent CNNs from performing long-range dependency modelling across the image, limiting the performance of CNN-based SR^18,19^. Recently, as an alternative to CNN, the Vision Transformer (ViT)^20,21^ demonstrated remarkable performance in addressing challenging tasks in the field of computer vision, owing to its ability to capture global interactions between contexts. In subsequent scholarly pursuits, researchers have further developed Transformer’s self-attention paradigm in diverse network designs for SR, notably by the works of SwinIR^22^, ELAN^23^, and HAT^24^. An essential component is popular Transformer-based SR methods is the window self-attention (W-SA) mechanism. By bringing locality into self-attention, the W-SA mechanism enhances the utilization of spatial information derived from input images.

While Transformer-based architectures have demonstrated strong performance on natural image SR tasks, their efficacy in remote sensing image SR remains inadequately explored and warrants systematic investigation. Compared to natural images, remote sensing imagery exhibits significantly greater complexity in terms of multi-scale spatial feature distribution and fine-grained information density. The substantial morphological variance and scale diversity of objects within such imagery present notable challenges for achieving high-fidelity reconstruction, particularly in high-frequency regions (e.g., shapes, contours, and edges). Most Transformer-based SR methodologies adopt W-SA mechanisms with rigidly constrained window sizes (e.g., 8$\times$8 in SwinIR), limiting the receptive filed to a single scale and preventing the network from gathering multi-scale information such as local textures and repetitive patterns. Additionally, the quadratic computational demand of Transformers relative to input resolution creates a scalability bottleneck for remote sensing SR tasks, where large-size reconstructions are often required.

**Fig. 1:**
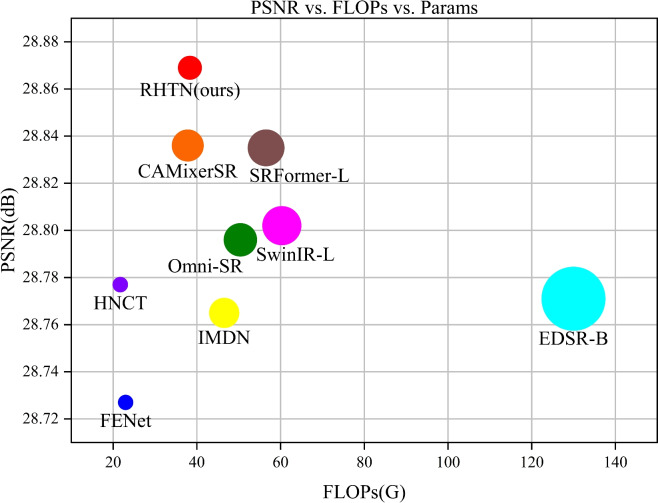
Model complexity and performance comparison between RHTN model and other SR methods on the RSSCN7 dataset for the magnification of $\times$4. Circle sizes indicate the number of parameters.

To address the aforementioned challenges, we propose a novel remote sensing images SR network called a residual hierarchical transformer network (RHTN), which achieves a comparable trade-off between computational complexity and SR reconstruction quality. The RHTN comprises three key components: shallow feature extraction, deep feature extraction, and reconstruction. During the deep feature extraction stage, a carefully designed residual hierarchical transformer block (RHTB) that was inspired by ViT architecture. In the RHTB, firstly, the conventional fixed windows used in self-attention computation are replaced with extended hierarchical windows to progressively enlarge the network’s receptive field and extract multi-scale features. Second, by simultaneously leveraging spatial attention and channel attention from both spatial and channel dimensions, the mechanism maximizes the learning of long-range dependencies in remote sensing images to restore texture details in reconstructed images. Furthermore, to alleviate the high computational complexity inherent in large-scale window self-attention, this invention employs only the query matrix and value matrix during attention computation, thereby reducing information redundancy caused by the key matrix. Finally, a spatial-gate feed-forward network (SGFN) is introduced to acquire more nonlinear features while reducing channel redundancy. Results as Fig. 1, our RHTN demonstrates outstanding efficiency compared to state-of-the-art approaches. In summary, the main contributions of this work are as follows:

1. To mitigate the high spatial and temporal complexity arising from the intricate architecture of Transformer-based models in image super-resolution tasks, we proposed the residual hierarchical transformer network (RHTN). This framework enables convenient and end-to-end trainable SR reconstruction of remote sensing imagery, achieving both high efficiency and superior reconstruction quality.
2. To cope with the increasing computational burdens of W-SA in handling large windows, we propose a novel spatial-channel self-attention mechanism that effectively integrates both spatial and channel-wise features while maintaining linear computational complexity relative to window dimensions. This approach facilitates the deployment of large-scale hierarchical window configurations in practical implementations.
3. We conduct experiments to confirm the effectiveness of the proposed method. The results of our experiments, conducted on three frequently used benchmark datasets (RSSCN7, WHURS19, and COWC), show that our method outperforms other state-of-the-art approaches in both quantitative metrics and visual outcomes.

The remainder of this paper is organized as follows. “Related work” introduces previous works on CNN-based and Transformer-based SR reconstruction algorithms, “Methodology” presents a detailed description of the RHTN, “Experiments and results” presents a verification of its effectiveness by experimental comparisons, and “Conclusions” concludes our work.

## Related work

### CNN-based SR

Since SRCNN^13^ pioneered the application of CNN to image SR, the filed has witnessed a proliferation of CNN-based architectures. To reduce computational consumption, Faster-SRCNN^13^ extracts features at the low-resolution (LR) scale and uses a deconvolution layer to perform an up-sampling operation at the end of the network. As with the excellent performance of classical ResNet^25^ in computer vision tasks, some early CNN-based methods (e.g., EDSR^16^, RDN^26^, RCAN^17^) attempt to deepen the layer of the network for better performance. For instance, RCAN^17^ implements a residual-in-residual architecture and constructs a deep network with over 400 layers.

While deeper networks can enhance performance, their increased parameter counts and computational demands pose significant challenges for deployment on resource-limited mobile devices. In response to this issue, several CNN-based methods are proposed to address SR in an efficient way. DRCN^27^ and DRRN^28^ employs recurrent block to decrease parameters, but suffered intensive computations. To mitigate these limitations, IDN^29^, IMDN^30^, and RFDN^31^ introduce an efficient information distillation mechanism, simultaneously reducing model parameters and computational costs. Recent approaches further enhance the computational efficiency of image SR by adopting network pruning^32,33^.

Recent studies employ well-designed attention mechanisms to suppress irrelevant information, such as channel attention^17^, pixel attention^34^, and holistic attention^35^. Besides, some researchers introduces similar attention^36^ and non-local sparse attention^37^ to extract global prior knowledge (e.g., self-similarity). However, restricted by the inherent limited receptive filed of CNNs, most CNN-based methods still struggle to effectively capture global dependencies in both spatial and channel dimensions.

### Transformer-based SR

Recently, Transformers have shown great potential in the field of natural language processing (NLP), has proven effective in modeling long-range dependencies and dynamically adjusting weights according to input characteristics. Consequently, Transformers have found widespread application in both high-level^38–40^ and low-level^41–43^ vision tasks. Vision Transformers (ViT)^21^ revolutionizes computer vision paradigms by reformulating image processing through a patch-based sequence representation. The subsequent development of Swin Transformer architecture^44^ achieved significant computational efficiency improvements via its innovative W-SA mechanism. For the SR task, the restored process requires preserving structural information in the input, posing a significant challenge for Transformer-based model design. IPT^45^ is a pre-trained model based on the standard transformer structure and has been improved the performance of low-level tasks, such as SR and denoising. SwinIR^43^ leverages the Swin Transformer^44^ encoder with spatial W-SA attention and shift operations, demonstrating the strong potential of Transformers for SR tasks. To address ViT’s high computational cost and GPU memory demands, ESRT^46^ captures long-range dependencies among similar patches. Compared to the original Transformer which occupied 16057M GPU memory, ESRT only occupies 4191M GPU memory.

Although Transformer can mitigates shortcomings of CNNs, its complexity is quadratic with the image resolution ($H\times W$), i.e., $\mathcal {O}\left. ((H W)^{2})\right.$, which limits its application to high-resolution (HR) image SR tasks. Moreover, current Transformer-based SR approaches with W-SA mechanism suffer from additional limitations as their fixed window size restricts multi-scale feature extraction, particularly for capturing local textures and repetitive patterns. In response to these challenges, Restormer^47^ employs cross-covariance computations across channel dimensions instead of spatial ones, making the complexity of SA linear with image resolution. DAT^48^ aggregates spatial and channel features in the inter-blok and intra-block for powerful representation competence. HAT^49^ introduces overlapping window cross-attention and hybrid attention blocks to effectively activate additional pixels in SR tasks. SRFormer^50^ establishes pairwise relationships with large windows (e.g., 24$\times$24) by transferring spatial information into the channel dimension.

These methods demonstrate Transformers’ superiority over CNNs while revealing two persistent challenges: computational complexity reduction and efficient extraction of spatial-channel information.

**Fig. 2:**
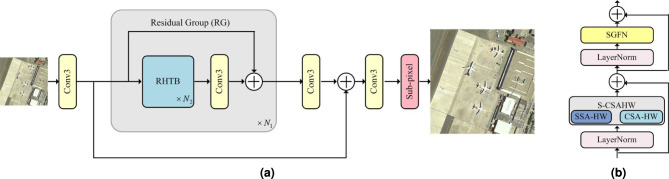
The network architecture of our method. **a** Residual hierarchical transformer network (RHTN); **b** Residual hierarchical transformer block (RHTB).

## Methodology

In this section, we will describe the proposed RHTN in detail. An overall graphical depiction of RHTN is shown in Fig. 2, which contains shallow feature extraction, deep feature extraction, and reconstruction. Here, we denote an initial LR input image and an SR output image as ${I}_{LR}$ and ${I}_{SR}$, respectively.

### Network architecture

The shallow feature extraction module adopts one convolutional layer with kernel 3$\times$3 to obtain shallow features ${F}_{0}\in {\mathbb {R}}^{H\times W\times C}$(*H*, *W* and *C* denote the image height, width, and channel number, respectively) from LR input images ${I}_{LR}\in {\mathbb {R}}^{H\times W\times 3}$ , the process can be represented as follows:

1 $$\begin{aligned} {F}_{0}={f}_{Conv3\times 3}({I}_{LR}) \end{aligned}$$

where ${f}_{Conv3\times 3}$ means the convolution operation. ${F}_{0}$ will be sent into the deep-level extraction module, the process can be represented as follows:

2 $$\begin{aligned} {F}_{DF}={H}_{DF}({F}_{0}) \end{aligned}$$

where ${H}_{DF}$ denotes the deep-level extraction module and it contains ${N}_{1}$ cascaded residual groups (RGs) and a convolutional layer with kernel 3$\times$3. More specifically, intermediate features ${F}_{1}$, ${F}_{2}$, $\cdots$, ${{F}_{N}}_{1}$ and the output deep feature ${F}_{DF}$ are extracted with a residual structure:

3 $$\begin{aligned} & {F}_{i}={{f}_{RG}}_{i}({{f}_{RG}}_{i-1}(\cdots ({{f}_{RG}}_{1}({F}_{0}))\cdots )),i=1,\cdots ,{N}_{1} \end{aligned}$$

4 $$\begin{aligned} & {F}_{DF}={f}_{Conv3\times 3}({{F}_{N}}_{1}) \end{aligned}$$

where ${{f}_{RG}}_{i-1}$ and ${{f}_{RG}}_{i}$ denote the *i*-th RG input and output, and ${f}_{Conv3\times 3}$ denotes the last convolutional layer. The use of a convolutional layer at the end of feature refinement can bring the inductive bias of the convolution operation into the Transformed-based method, and provide a better foundation for the later aggregation of shallow and deep features. As shown in Fig. 2, the RG is stacked by ${N}_{2}$ residual hierarchical transformer block (RHTB) and a convolutional layer with kernel 3$\times$3. Given the input feature ${F}_{i,0}$ of the *i*-th RG, intermediate features ${F}_{i,1}$, ${F}_{i,2}$, $\cdots$, ${F}_{i,{N}_{2}}$ and the output deep feature ${F}_{i,out}$ are extracted as follows:

5 $$\begin{aligned} & {F}_{i,j}={{f}_{RHTB}}_{i,j}({{f}_{RHTB}}_{i,j-1}(\cdots ({{f}_{RHTB}}_{i,1}({F}_{i,0}))\cdots )),i=1,\cdots ,{N}_{1};j=1,\cdots ,{N}_{2} \end{aligned}$$

6 $$\begin{aligned} \ & {F}_{i,out}={f}_{Conv3\times 3}({{F}_{i,{N}_{2}}})+({{F}_{i,0}}) \end{aligned}$$

where ${{f}_{RHTB}}_{i,j-1}$ and ${{f}_{RHTB}}_{i,j}$ denote the *j*-th RHTB input and output in the *i*-th RG, and ${f}_{Conv3\times 3}$ denotes the convolutional layer in the *i*-th RG.

The summation results of ${F}_{0}$ and ${F}_{DF}$ are fed into the reconstruction module for HR image reconstruction. Firstly, a global residual connection links the shallow features to the output of the deep features for capturing the high-frequency details. Then, a convolutional layer with kernel 3$\times$3 is utilized to aggregate the features. After that, a sub-pixel convolutional layer^51^ is utilized to upsample the deep features to the same size of the HR output. The process can be represented as follows:

7 $$\begin{aligned} {I}_{SR}={f}_{Subpixel}({f}_{Conv3\times 3}({F}_{0}+{F}_{DF})) \end{aligned}$$

where ${f}_{Conv3\times 3}$ denotes the convolution operation, ${f}_{Subpixel}$ denotes the sub-pixel convolution operation, the SR output ${I}_{SR}\in {\mathbb {R}}^{sH\times sW\times 3}$, where *s* denotes the upscaling factor. Network parameter optimization is performed using the L1 loss:

8 $$\begin{aligned} \mathcal {L}={\left\| {I}_{SR}-{I}_{HR} \right\| }_{1} \end{aligned}$$

where ${I}_{HR}$ denotes the corresponding ground-truth HR image.

### The residual hierarchical transformer block (RHTB)

The deep feature extraction module is designed to capture complex information from images with large variations in target feature scales. The proposed stage includes a novel residual hierarchical transformer block (RHTB) module, as depicted in Fig. 1, which is more suitable for remote sensing SR tasks than the previous Transformer structures^50,52^. Taking inspiration from ViT network, which leverage self-attention for global feature aggregation and feed-forward network for feature refinement, the RHTB formulate the spatial-channel self-attention based on hierarchical windows (S-CSAHW) and spatial-gate feed-forward network (SGFN) into a unified feature mixing module designed for representative feature selection. The feature mixing module can be formulated as:

9 $$\begin{aligned} & Y=S-CSAHW(LN(X))+X \end{aligned}$$

10 $$\begin{aligned} & Z=SGFN(LN(Y))+Y \end{aligned}$$

where *LN* denotes the LayerNorm^21^ layers, *X*, *Y*, and *Z* denote the intermediate features. Next, we provide a detailed description of S-CSAHW and SGFN below.

### The spatial-channel self-attention based on hierarchical windows (S-CSAHW)

The standard Transformer^43^ takes a set of 1-D sequences of token embedding as input. To handle 3-D features of size $H\times W\times C$, we reshape the input to a $\frac{HW}{hw}\times N\times C$ feature by partitioning the input into non-overlapping $h\times w$ local windows, where $N=\frac{HW}{hw}$ is the resulting number of windows and also is the effective input sequence for the Transformer. For a local window feature $X\in {\mathbb {R}}^{h\times w\times C}$, the corresponding *query*, *key*, and *value* matrices *Q*, *K*, and *V* are computed as:

11 $$\begin{aligned} Q=X{P}_{Q},K=X{P}_{K},V=X{P}_{V} \end{aligned}$$

where ${P}_{Q}$, ${P}_{K}$, and ${P}_{V}$ denote the weight matrices that are shared across windows. By comparing the similarity between *Q* and *K*, we obtain an attention map and multiply it with *V*. Overall, the calculation of W-SA can be expressed as follows:

12 $$\begin{aligned} Attention(Q,K,V)=Softmax(Q{K}^{T}/\sqrt{d}+B)\cdot V \end{aligned}$$

where *B* denotes learnable relative positional encoding, and *d* denotes the dimension of the *Q* or *V* matrices. Compared with CNN-based methods, the Windowed W-SA mechanism can effectively establish global context information in remote sensing images^53,54^, thereby overcoming the limitation of convolutional receptive fields. However, the vectorization of 2-D images can generate excessively high-dimensional feature vectors, consequently elevating computational demand. Moreover, conventional self-attention mechanisms compute interactions solely between *Q* and *K* vectors while ignoring the potentially informative interactions between queries and keys.

Similar to the interaction between *Q* and *K*, an intrinsic correlation exists between *Q* and *V*. Incorporating *Q*-*V* interactions can enhance the output through customized feature learning based on their characteristics. As demonstrated in Work^55^, introducing such interactions significantly strengthens the semantic representation capacity of traditional self-attention mechanisms. In RHTB, unlike standard W-SA methods that predict *Q*, *K*, and *V* by linear projection, we equate keys with values as they both reflect the intrinsic properties of input features, and only estimate queries $Q\in {\mathbb {R}}^{H\times W\times \frac{C}{2}}$ and values $V\in {\mathbb {R}}^{H\times W\times \frac{C}{2}}$. Then we partition queries and keys to non-overlapped windows according the the assigned window size, e.g., ${Q}_{i}$ and ${V}_{i}$ for the *i*-th RHTB, and use the partitioned queries and values for the subsequent correlation mechanism.

**Fig. 3:**
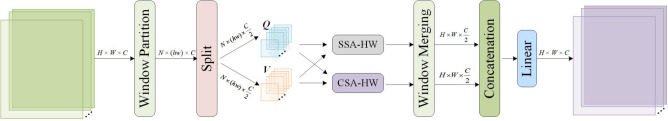
The operational procedure of S-CSAHW.

Following feature fusion, the resulting *Q* and *V* are fed into spatial self-attention with hierarchical windows (SSA-HW) and channel self-attention with hierarchical windows (CSA-HW) to extract multi-scale spatial and channel features. The process can be formulated as:

13 $$\begin{aligned} {F}_{SAHW}={H}_{WM}({H}_{SAHW}(Q,V)),{F}_{CAHW}={H}_{WM}({H}_{CAHW}(Q,V)) \end{aligned}$$

where, ${H}_{SAHW}$ and ${H}_{CAHW}$ denote the operations of SSA-HW and CSA-HW, respectively; ${H}_{WM}$ denotes the window merging process, which is applied after calculating attention values at specific scales; ${F}_{SAHW},{F}_{CAHW}\in {\mathbb {R}}^{H\times W\times \frac{C}{2}}$ denote the spatially and channel-wise features hierarchically extracted from these windows. After that, features ${F}_{SAHW}$ and ${F}_{CAHW}$ undergo channel-wise concatenation, followed by a feature linear transformation on the merged tensor. This transformed representation serves as the final output of the S-CSAHW module, formulated as:

14 $$\begin{aligned} {F}_{S-CAHW}={f}_{Linear}({f}_{concat}({F}_{SAHW},{F}_{CAHW})) \end{aligned}$$

where, ${f}_{Linear}$ denotes a fully-connected layer preserving feature channel dimensionality, ${f}_{concat}$ denotes the feature-map concatenation operation, and ${F}_{S-CAHW}$ denotes the output representation of the S-CSAHW module. Fig. 3 illustrates the detailed operational procedure of S-CSAHW.

#### *SSA-HW*

Current Transformer-based SR approaches^43,46^ often employ W-SA with fixed small window sizes, e.g., 8$\times$8 in SwinIR^43^, limiting the receptive filed to a single scale and preventing the network from gathering multi-scale information such as local textures and repetitive patterns. In addition, the quadratic computational complexity of W-SA to the window size also makes the expansion of receptive fields unaffordable in practice. To mitigate the computational overhead, some previous approaches have adopted the strategy of reducing channels to provide a larger receptive field (e.g., 16$\times$16 in ELAN^56^ and 24$\times$24 in SRFormer^50^). However, these methods not only suffer from trade-off between spatial and channel information but also remain quadratic complexity to window sizes. Therefore, how to effectively aggregate multi-scale features while maintaining computational efficiency remains a critical problem for transformer-based SR approaches.

**Fig. 4:**
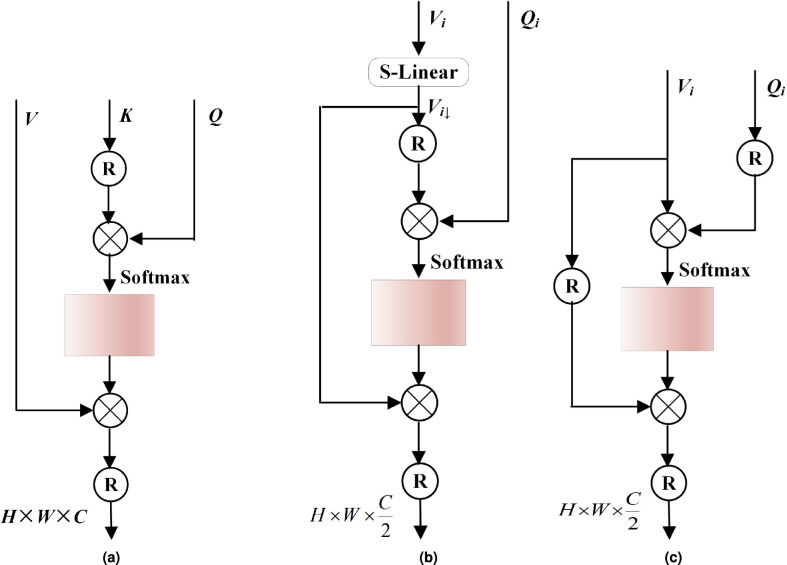
Structure of **a** W-SA, **b** SSA-HW, and **c** CSA-HW.

As shown in Fig. 4b, we denote the reshaped projection matrices as ${Q}_{i}$ and ${V}_{i}$ (all sizes are $\in {\mathbb {R}}^{N\times {h}_{i}{w}_{i}\times \frac{C}{2}}$) in *i*-th RHTB. Then, we split them into *M* heads: ${Q}_{i}=[{Q}_{i}^{1},\cdots ,{Q}_{i}^{M}]$ and ${V}_{i}=[{V}_{i}^{1},\cdots ,{V}_{i}^{M}]$. The dimension of each head is $d=\frac{C}{M}$ . Considering the expanding window sizes in hierarchical strategy, we first adaptively summarize the spatial information of values ${V}_{i}\in {\mathbb {R}}^{hw\times \frac{C}{2}}$ in different RHTBs by applying linear layers on the spatial dimension:

15 $$\begin{aligned} {V}_{i\downarrow }={f}_{Linear-i}({V}_{i}) \end{aligned}$$

where ${f}_{Linear-i}$ denotes a fully-connected layer adaptively summarizing the spatial information, and ${V}_{i\downarrow }\in {\mathbb {R}}^{hw\times \frac{C}{2}}$ denote the projected values with:

16 $$\begin{aligned} {h}_{i\downarrow }={\left\{ \begin{array}{ll} {\alpha }_{i}{h}_{Base},& if={\alpha }_{i}\leqslant 1\\ {h}_{Base},& if={\alpha }_{i}> 1 \end{array}\right. },{w}_{i\downarrow }={\left\{ \begin{array}{ll} {\alpha }_{i}{w}_{Base},& if={\alpha }_{i}\leqslant 1\\ {w}_{Base},& if={\alpha }_{i}> 1 \end{array}\right. } \end{aligned}$$

where: ${h}_{Base}$ and ${w}_{Base}$ denote the height and width of the default base window (typically configured as 8$\times$8), respectively; ${\alpha }_{i}$ denotes the hierarchical ratio in *i*-th RHTB. Thereby, the architecture simultaneously aggregates high-level semantics from large-scale receptive fields while preserving fine-grained local patterns through small-window operations. The attention matrix is thus computed by the self-attention mechanism in a local window as:

17 $$\begin{aligned} {Y}_{i}^{m}=Softmax({Q}_{i}^{m}({{V}_{i\downarrow }^{m}})^{T})/\sqrt{d}+B)\cdot {{V}_{i\downarrow }^{m}} \end{aligned}$$

where: *B* denotes the relative position encoding^20^, ${Y}_{i}^{m}\in {\mathbb {R}}^{{h}_{i\downarrow }\times {w}_{i\downarrow }\times d}$ denotes the *i*-th RHTB attention feature in the *m*-th head. Finally, we obtain the features ${Y}_{i}\in {\mathbb {R}}^{H\times W\times \frac{C}{2}}$ by reshaping and concatenating all ${Y}_{i}^{m}$. The process is formulated as:

18 $$\begin{aligned} & {Y}_{i}={f}_{concat}({Y}_{i}^{1},\cdots ,{Y}_{i}^{M}) \end{aligned}$$

19 $$\begin{aligned} & SSA-HW(X)={Y}_{i}{W}_{p} \end{aligned}$$

where ${W}_{p}\in {\mathbb {R}}^{\frac{C}{2}\times \frac{C}{2}}$ denotes the projection matrix for feature fusion. Compared with the standard W-SA, the proposed SSA-HW reduces the computational complexity of self-attention per window from $\mathcal {O}(C{({h}_{i}{w}_{i})}^{2})$ to $\mathcal {O}(C{h}_{i\downarrow }{w}_{i\downarrow }{h}_{i}{w}_{i})$. Specifically, supposing the input sequences consisting of *N* windows, each with space dimensions ${\mathbb {R}}^{{h}_{i}{w}_{i}\times C}$, the numbers of mult-add operations required for W-SA and SSA-HW can be calculated as follows:

20 $$\begin{aligned} & Mult-Add(W-SA)=2NC({h}_{i}{w}_{i})^{2} \end{aligned}$$

21 $$\begin{aligned} & Mult-Add(SSA-HW)=2NC{h}_{i\downarrow } {w}_{i\downarrow }{h}_{i}{w}_{i} \end{aligned}$$

where the former exhibits quadratic complexity with respect to the hierarchical window size ${h}_{i}{w}_{i}$. In contrast, the computational complexity of our SSA-HW scales linearly with ${h}_{i}{w}_{i}$, as the size ${h}_{i\downarrow }{w}_{i\downarrow }$ are constrained by the fixed base window ${h}_{B}{w}_{B}$. This linear complexity advantage enables efficient window scaling, allowing SSA-HW to aggregate features across multiple windows while expanding the effective attention region without incurring prohibitive computational costs.

#### *CSA-HW*

Apart from spatial information, we further design CSA-HW to gather features along the channel dimension, as depicted in Fig. 4c. Same as the operation in SSA-HW, we denote the reshaped projection matrices as ${Q}_{i}$ and ${V}_{i}$ as the input of CSA-HW in the *i*-th RHTB. It should be noted that unlike SSA-HW, the CSA-HW adopts a single-head strategy in contrast to multi-head approach. This design choice stems from an important observation: the multi-head mechanism inherently constrains the receptive field for channel-wise information aggregation, wherein each channel can only establish interactions within a restricted subset of other channels^5^. Such constrained cross-channel communication leads to suboptimal feature integration. By employing a single-head architecture, CSA-HW facilitates comprehensive inter-channel interactions across the entire feature space, thereby overcoming this fundamental limitation. The process of CSA-HW is formulated as:

22 $$\begin{aligned} CSA-HW(X)=Softmax(({Q}_{i})^{T}{V}_{i}/\sqrt{d}_{i})\cdot {{V}_{i}^{T}} \end{aligned}$$

where ${d}_{i}={h}_{i}{w}_{i}$. In contrast to prevalent transposed attention mechanisms for channel aggregation^47,48^, our proposed CSA-HW architecture achieves the following innovations: (1) hierarchical window-based processing that systematically captures spatial-contextual relationships, and (2) effective exploitation of multi-scale feature representations to enhance super-resolution reconstruction fidelity. For the *i*-th RHTB, the per-window channel attention computational cost is linear in window size $\mathcal {O}((C)^{2}{{h}_{i}{w}_{i}})$. Similarly, supposing the input sequences with each hierarchical window in the ${\mathbb {R}}^{{h}_{i}{w}_{i}\times C}$ space, the numbers of mult-add operations required for CSA-HW can be calculated as follows:

23 $$\begin{aligned} Mult-Add(CSA-HW)=2NC^{2}{h}_{i}{w}_{i} \end{aligned}$$

Therefore, the proposed CSA-HW can enable scalable windows to facilitate comprehensive hierarchical feature integration across network stages.

**Fig. 5:**
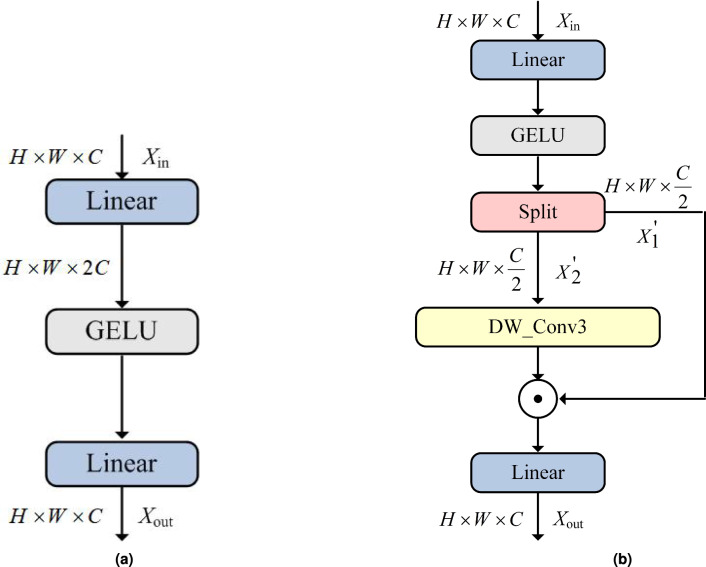
Structure of **a** FFN and **b** SGFN.

### The spatial-gate feed-forward network (SGFN)

Next, following completion of window-based attention computation, a SGFN^48^ is employed to enhance representational capacity through learnable linear transformations and non-linear activation functions. The MLP-based feed-forward network (FFN)^20^ consists of two linear transformations with a RELU activation to extract features. The deficiency of FFN lies in its capability to merely model feature channels while ignoring the modeling of spatial information. Moreover, FFN internally amplifies the number of the feature channels through the linear projection layer, resulting in redundancy between channels, which hampers the feature representation capability. Compared with FFN, the SGFN is able to capture non-liner spatial information and ease the channel redundancy of fully-connected layers by introducing a simple gate mechanism.

To be specific, when provided with an input tensor ${X}_{in}\in {\mathbb {R}}^{H\times W\times C}$, the SGFN can be expressed as:

24 $$\begin{aligned} & {X}_{1}^{'},{X}_{2}^{'}={f}_{split}({f}_{GELU}({W}_{lp}^{1}({X}_{in}))) \end{aligned}$$

25 $$\begin{aligned} & {X}_{out}={W}_{lp}^{2}({X}_{1}^{' }\odot ({W}_{d}({X}_{2}^{' }))) \end{aligned}$$

where: ${f}_{split}$ denotes the feature-channel splitting operation; ${W}_{d}$ denotes the learnable parameters of the depth-wise convolution, which can maintain computational efficiency; ${W}_{lp}^{1}$ and ${W}_{lp}^{2}$ denote linear projection operations with channel transformations C$\rightarrow$C and C/2$\rightarrow$C repectively. Fig. 5b illustrates the structure of SGFN.

## Experiments and results

In this section, firstly, we demonstrate the experimental settings, including datasets, evaluation metrics, and training implementation details. Then, we report the experimental results and the correlation analysis.

### Experimental settings

#### *Dataset settings and evaluation*

Following previous work^57,58^, we used the recently popular Aerial Image Dataset (AID)^59^ as the training data set, which is a collection of remote sensing images depicting 30 land-use classes, including airport, bare land, baseball field, beach, bridge, center, church, commercial, dense residential, desert, farmland, forest, industrial, meadow, medium residential, mountain, park, parking, playground, pond, port, railway station, resort, river, school, sparse residential, square, stadium, storage tanks, and viaduct. In all, the AID dataset has the number of 10000 images with a pixel size of 600$\times$600. When testing our method, we assess its performance on theree public datasets, as follows:

1. RSSCN7^60^: The RSSCN7 consists of a total of 2800 remote sensing images from Google Earth, covering seven classic remote sensing scenarios, including grasslands, fields, factory areas, lakes, forests, residential areas, and parking lots. Each scenario contains 400 images with a pixel size of 400$\times$400.
2. WHU-RS19^61^: The WHU-RS19 dataset mainly comes from remote sensing images obtained from Google satellites, covering 19 categories of the physical scenes,including airport, beach, bridge, commercial area, desert, farmland, football field, forest, industrial area, meadow, mountain, park, parking, pond, port, railway station, residential area, river, and viaduct. Each category contains an average of about 50 images with a pixel size of 600$\times$600.
3. COWC^62^: The COWC (Cars Overhead with Context) dataset contains a large number of unique cars from six different images sets each covering a different geographical location and produced by different images. The images cover regions from Toronto in Canada, Selwyn in New Zealand, Potsdam and Vaihingen in Germany, Columbus and Utah in the United States. In this paper, we chosen 3500 image tiles with a pixel size of 256$\times$256 to ensure the diversity of background coverage.

All LR images were obtained by downsampling the corresponding HR label samples through Bicubic interpolation with $\times$2, $\times$3, and $\times$4 scale factors. In the training phase, patches with a size of 48$\times$48 were randomly cropped from LR images and the reference patches from their corresponding HR ones as the input of the model. To fully utilize the samples, the training dataset was augmented via three image-processing methods: (1) horizontal flipping; (2) vertical flipping; and (3) $90^\circ$rotation.

To objectively evaluate the quality of the restored remote sensing images, the two widely-used image assessment metrics are used here: the peak signal-to-noise ratio (PSNR) and the structural similarity index (SSIM)^63^. All PSNR and SSIM values are calculated on the Y channel (i.e., luminance) of images transformed to YCbCr color space. Furthermore, to better demonstrate the perceptual quality of our model, we additionally adopt the learned perceptual image patch similarity (LPIPS) metric for comparison with state-of-the-art methods. In LPIPS, lower values indicate higher perceptual similarity between images, while higher values correspond to greater perceptual differences.

#### *Implementation details*

All experiments are conducted with the Pytorch framework on NVIDIA RTX 4090 GPUs. The training iterations are 500K with a batchsize of 16. We set the initial learning rate to $2\times {10}^{-4}$, and half it an milestones: [250K, 400K, 450K, 475K]. The SR network was optimized with Adam^64^ by setting ${\beta }_{1}=0.9$, ${\beta }_{2}=0.999$, and $\epsilon ={10}^{-8}$.

In RHTN, we set the RG number as ${N}_{1}$=4 and the RHTB number as ${N}_{2}$=6 for each RG. In RHTB, the channel dimension, base window size and attention number head are set as 60, 8 and 6, respectively. The hierarchical ratios are set as [0.5, 1, 2, 4, 6, 8] for the 6 RHTBs in each RG. In SGFN, the input feature dimension and hidden dimension performed by first linear projection are set as 60 and 120, respectively.

### Ablation study

In this section, this study conduct a series of experiments on the WHURS19 test dataset to explore the importance of each component in proposed method, where all models are trained with the same settings. For simplicity, these experiments are carried out with a magnification of $\times$4.

**Table 1:** Ablation experiments on architecture variants of RHTB. Bold indicates the optimal performance.

Variants	Params	PSNR	SSIM
SSA-HW $\rightarrow$ None	521K	30.761	0.8066
CSA-HW $\rightarrow$ None	524K	30.719	0.8054
*Q*, *V* $\rightarrow$ $Q$, *K*, *V*	740K	30.780	0.8081
SGFN $\rightarrow$ None	286K	30.525	0.7992
RHTN	567K	**30.808**	**0.8084**

#### *Effects of RHTB*

The Effects of S-CSAHW:

The present S-CSAHW integrates both spatial and channel-wise feature representations by leveraging hierarchical self-attention between queries and values. S-CSAHW consists of two main modules: SSA-HW and CSA-HW. To validate the necessity of these components, we conduct experiments by replacing S-CSAHW with only SSA-HW or CSA-HW. As shown in Table 1, without SSA-HW, the PSNR and SSIM values on the test dataset will drop by 0.047 dB and 0.0018, respectively. Without CSA-HW, the PSNR and SSIM values on the test dataset will drop by 0.089 dB and 0.0030, respectively. It is obvious that SSA-HW significantly outperforms CSA-HW, while RHTN achieves the best overall performance by effectively leveraging both spatial and channel information. Furthermore, we construct a variant using standard queries-keys-values (*QKV*) computation^43^ for comparison. As shown in Table 1, the *QKV* configuration achieves comparable accuracy to our *QV* variant while exhibiting significantly higher computational costs (Params: $\uparrow$173K). This efficiency gap arises because both keys and values encode overlapping intrinsic features of the input, leading to redundant computations. By eliminating the key projection while retaining queries and values, the proposed *QV* variant reduces this redundancy without compromising performance.

**Table 2:** Ablation experiments on different window arrangement strategies of S-CSAHW. Bold indicates the optimal performance.

Strategies	Params	PSNR	SSIM
Windows = [8, 8, 8, 8, 8, 8]	566K	30.763	0.8069
Windows = [64, 64, 64, 64, 64, 64]	568K	30.770	0.8074
Windows = [64, 48, 32, 16, 8, 4]	567K	30.772	0.8077
Windows = [4, 8, 16, 32, 48, 64]	567K	**30.808**	**0.8084**

To verify the effect of our window strategy, we conduct experiments with different window arrangement methods, including fixed small windows (Windows = [8, 8, 8, 8, 8, 8]), fixed large windows (Windows = [64, 64, 64, 64, 64, 64]), and shrinking hierarchical windows (Windows = [64, 48, 32, 16, 8, 4]). As show in Table 2, models with fixed small windows can only exploit local features of SR, leading to sub-optimal performance. In contrast, models with fixed large windows establish long-range dependencies, thereby improving performance (PSNR: $\uparrow$0.007 dB, SSIM: $\uparrow$0.0005)–yet at the cost of increased computational overhead. Compared to fixed-window methods, the hierarchical-windows approaches effectively capture multi-scale semantic information across deep network layers, thereby achieving a better balance between super-resolution performance and model complexity. Furthermore, compared to shrinking hierarchical windows, the expanding hierarchical windows adopted in RHTN achieves better performance (PSNR: $\uparrow$0.036 dB, SSIM: $\uparrow$0.0007). This improvement stems from the extended window strategy’s ability to first utilize small windows for extracting the most relevant features, followed by gradually expanding larger windows to establish reliable long-range dependencies.

**Table 3:** Ablation experiments on different self-attention head strategies of CSA-HW. Bold indicates the optimal performance.

Strategies	Params	PSNR	SSIM
head = 6	567K	30.772	0.8056
head = 1	567K	**30.808**	**0.8084**

Unlike the common multi-head strategy employed in self-attention calculations for SSA-HW, CSA-HW adopts a single-head approach to efficiently extract and fuse channel-wise information. To validate the effectiveness of the single-head strategy (head=1) in CSA-HW, we conduct an ablation study by comparing it with a conventional multi-head configuration (head=6), with results presented in Table 3. Compared to multi-head strategy, the single-head in CSA-HW enhances channel-wise information interaction, improving SR performance (PSNR: $\uparrow$0.036 dB, SSIM: $\uparrow$0.0007). This is because while the multi-head strategy effectively aggregates features from distinct channel sub-spaces in spatial self-attention computations, it inherently obstructs full channel-wise interactions during channel self-attention due to channel splitting.

The Effects of SGFN:

The SGFN integrates nonlinear spatial features through its gated feed-forward layers. To validate the necessity of SGFN, we first perform an ablation study by removing this module from RHTN for comparative analysis. As shown in Table 1, without SGFN, the PSNR and SSIM values will drop by 0.283 dB and 0.0092, respectively. Furthermore, we conduct an ablation study comparing SGFN with FFN^20^ to better demonstrate the superiority in Transformer-based SR networks, with results presented in Table 4. Compared to FFN, SGFN achieves both parameter reduction (Params: $\downarrow$69K) while maintaining superior performance (PSNR: $\uparrow$0.052 dB, SSIM: $\uparrow$0.0018).

**Table 4:** Ablation experiments of SGFN. Bold indicates the optimal performance.

Strategies	Params	PSNR	SSIM
FFN	636K	30.756	0.8066
SGFN	567K	**30.808**	**0.8084**

#### *Ablation study of RHTN*

**Fig. 6:**
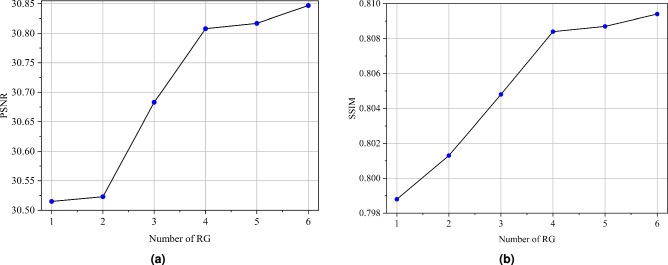
Performance comparison for RHTN with different numbers of RG.

We also perform ablation experiments on the whole RHTN, mainly including the number of RG and the number of layers of convolution. These experiments were designed to rigorously analyze the respective impacts of network depth and feature dimensionality on overall model performance. To verify the influence of using different numbers of RG in RHTN, we train models with different depths (${N}_{1}$ = 1, 2, 3, 4, 5, and 6), and a corresponding quantitative comparison of the results is presented respectively in Table 5 and Fig. 6. It is observed that the PSNR and SSIM results exhibit a generally positive correlation with the number of RG. While PSNR and SSIM metrics demonstrate an overall positive correlation with RG count, the performance gains diminish significantly at higher RG quantities. Specifically, the PSNR improvement decreases from 0.125 dB (${N}_{1}$: 3$\rightarrow$4) to 0.009 dB (${N}_{1}$: 4$\rightarrow$5), and the SSIM enhancement drops from 0.0056 to 0.003. Concurrently, the model’s parameter count inevitably escalates with additional RGs. Our proposed RHTN (${N}_{1}$=4) represents a balanced compromise between performance and computational efficiency.

**Fig. 7:**
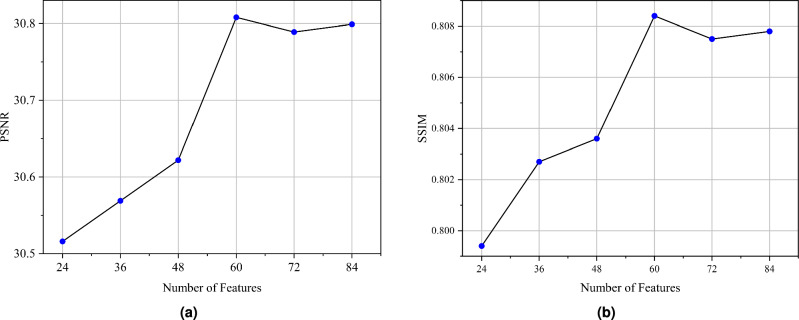
Performance comparison for RHTN with different numbers of Features.

Furthermore, we increase the number of features in the model, gradually increasing from the 24 to 84, and the corresponding quantitative comparison of the results is presented respectively in Table 6 and Fig. 7. Experimental results demonstrate that the SR effect of the model does not continuously improve with in the number of features. While increasing feature channels from 48 to 60 enhanced SR performance (PSNR: $\uparrow$0.186 dB, SSIM: $\uparrow$0.0048), extending beyond 60 channels led to diminished results (PSNR: $\downarrow$0.019 dB, SSIM: $\downarrow$0.0009). These findings suggest that 60 feature channels represent a balanced configuration, simultaneously minimizing model complexity while maintaining superior reconstruction performance.

**Table 5:** Ablation experiments on different RG quantities in RHTN. Bold indicates the optimal performance.

Number of RG	Params	PSNR	SSIM
1	187K	30.515	0.7988
2	314K	30.523	0.8013
3	440K	30.683	0.8048
4	567K	30.808	0.8084
5	694K	30.817	0.8087
6	820K	**30.847**	**0.8094**

**Table 6:** Ablation experiments on the number of layers of convolution in RHTN. Bold indicates the optimal performance.

Number of Features	Params	PSNR	SSIM
24	105K	30.516	0.7994
36	219K	30.569	0.8027
48	373K	30.622	0.8036
60	567K	**30.808**	**0.8084**
72	802K	30.789	0.8075
84	1079K	30.799	0.8078

### Comparisons with other methods

We present quantitative results for $\times$2, $\times$3, and $\times$4 SR, comparing against current efficient state-of-the-art approaches, including Bicubic, EDSR^16^, IMDN^30^, SwinIR-L^43^, HNCT^65^, FENet^66^, Omni-SR^67^, SRFormer-L^50^, and CAMixerSR^52^. Among them, SwinIR-L, HNCT, Omni-SR, SRFormer-L, and CAMixerSR are Transformer-based image SR approaches. Table 7 presents a comparison of the latency and performance accuracy of various methods on the test dataset.

**Table 7:** Comparison of quantitative results of different methods on test datasets. Bold indicates the optimal performance, and underlining indicates the second-best performance.

Method	Scale	Params	FLOPs	RSSCN7			WHU-RS19			COWC		
				PSNR	SSIM	LPIPS	PSNR	SSIM	LPIPS	PSNR	SSIM	LPIPS
Bicubic	$\times$2	–	–	31.640	0.8535	0.2749	35.010	0.9212	0.1515	32.215	0.8772	0.2725
EDSR-B	$\times$2	1370K	89.96G	32.941	0.8840	0.2117	36.612	0.9425	0.1172	34.165	0.8970	0.2096
IMDN	$\times$2	694K	45.19G	32.932	0.8838	0.2100	36.611	0.9422	0.1183	34.228	0.8974	0.2006
SwinIR-L	$\times$2	910K	59.04G	32.961	0.8845	0.2112	36.625	0.9426	0.1167	34.233	0.8975	0.2079
HNCT	$\times$2	357K	20.62G	32.933	0.8843	0.2077	36.462	0.9424	0.1178	34.159	0.8972	0.2013
FENet	$\times$2	351K	21.95G	32.892	0.8830	0.2139	36.582	0.9418	0.1192	34.128	0.8962	0.2083
Omni-SR	$\times$2	772K	49.04G	32.983	0.8849	0.2084	36.672	0.9428	0.1210	34.255	0.8979	0.1949
SRFormer-L	$\times$2	853K	55.26G	33.105	**0.8882**	**0.2000**	36.815	0.9445	0.1216	34.408	0.9017	0.1943
CAMixerSR	$\times$2	746K	36.34G	33.091	0.8876	0.2042	36.817	0.9443	0.1155	34.412	0.9015	0.1949
RHTN (ours)	$\times$2	547K	37.06G	**33.127**	0.8876	0.2027	**36.854**	**0.9447**	**0.1144**	**34.446**	**0.9019**	**0.1932**
Bicubic	$\times$3	–	–	29.199	0.7521	0.4586	31.295	0.8278	0.3009	30.039	0.8064	0.4425
EDSR-B	$\times$3	1555K	102.6G	30.200	0.7913	0.3804	32.744	0.8697	0.2357	31.415	0.8315	0.3816
IMDN	$\times$3	703K	45.75G	30.211	0.7916	0.3717	32.785	0.8696	0.2281	31.488	0.8326	0.3677
SwinIR-L	$\times$3	918K	59.57G	30.256	0.7931	0.3623	32.752	0.8707	0.2225	31.543	0.8334	0.3515
HNCT	$\times$3	363K	21.07G	30.226	0.7927	0.3604	32.772	0.8700	0.2219	31.499	0.8328	0.3600
FENet	$\times$3	357K	22.38G	30.159	0.7899	0.3752	32.702	0.8679	0.2299	31.395	0.8306	0.3754
Omni-SR	$\times$3	780K	49.60G	30.239	0.7932	0.3812	32.831	0.8713	0.2271	31.511	0.8335	0.3812
SRFormer-L	$\times$3	861K	55.79G	30.312	**0.7966**	**0.3591**	32.909	0.8736	**0.2212**	31.602	0.8362	**0.3507**
CAMixerSR	$\times$3	754K	37.55G	30.313	0.7960	0.3620	32.943	0.8733	0.2235	31.615	**0.8363**	0.3579
RHTN (ours)	$\times$3	556K	37.59G	**30.328**	**0.7966**	0.3600	**32.962**	**0.8738**	0.2216	**31.621**	**0.8363**	0.3515
Bicubic	$\times$4	–	–	27.896	0.6787	0.5589	29.301	0.7483	0.4188	27.354	0.7227	0.5375
EDSR-B	$\times$4	1518K	129.97G	28.771	0.7228	0.4510	30.561	0.8022	0.3137	30.074	0.7864	0.4494
IMDN	$\times$4	715K	46.54G	28.765	0.7227	0.4502	30.599	0.8018	0.3132	30.080	0.7864	0.4452
SwinIR-L	$\times$4	930K	60.31G	28.802	0.7244	0.4416	30.619	0.8034	0.3077	30.110	0.7873	0.4381
HNCT	$\times$4	373K	21.69G	28.777	0.7229	0.4535	30.586	0.8018	0.3164	30.014	0.7853	0.4587
FENet	$\times$4	366K	22.97G	28.727	0.7204	0.4569	30.526	0.7989	0.3196	29.980	0.7844	0.4560
Omni-SR	$\times$4	792K	50.40G	28.796	0.7246	0.4635	30.644	0.8042	0.3173	30.116	0.7877	0.4408
SRFormer-L	$\times$4	873K	56.53G	28.835	0.7278	**0.4379**	30.725	0.8071	**0.3054**	30.151	0.7898	**0.4328**
CAMixerSR	$\times$4	765K	37.82G	28.836	0.7267	0.4463	30.746	0.8062	0.3110	30.160	0.7896	0.4492
RHTN (ours)	$\times$4	567K	38.33G	**28.869**	**0.7285**	0.4400	**30.808**	**0.8084**	0.3058	**30.223**	**0.7911**	**0.4328**

#### *Quantitative results*

1. Quantitative Results on RSSCN7 Dataset: Taking the RSSCN7 dataset as a case study, it can be observed that RHTN obtain the highest value in terms of PSNR. Specifically, compared with other approaches, the average PSNR value of RHTN at the three magnifications is 1.196 dB higher than Bicubic interpolation, 0.137 dB higher than EDSR-B, 0.139 dB higher than IMDN, 0.102 dB higher than SwinIR-L, 0.129 dB higher than HNCT, 0,182 dB higher than FENet, 0.102 dB higher than Omni-SR, 0.024 dB higher than SRFormer-L, 0.028 dB higher than CAMixerSR. For SSIM, the average performance of RHTN is 0.0428 higher than Bicubic interpolation, 0.0049 higher than EDSR-B, 0.0049 higher than IMDN, 0.0036 higher than SwinIR-L, 0.0043 higher than HNCT, 0,0065 higher than FENet, 0.0037 higher than Omni-SR, 0.0001 higher than SRFormer-L, and 0.0007 higher than CAMixerSR. While RHTN demonstrates less significant performance improvements compared to SRFormer-L and CAMixerSR, it is noteworthy that RHTN maintains only 64.5% and 73.8% of the average model parameters of SRFormer-L and CAMixerSR, respectively, across three upscaling factors. For LPIPS, RTHN also demonstrates competitive parameter efficiency, with its average score closely approaching that of the best-performing SRFormer-L.
2. Quantitative Results on WHU-RS19 Dataset: Taking the WHU-RS19 dataset as an example. Unlike the RSSCN7 dataset, this dataset is substantially larger in scale and encompasses a broader range of scene categories totaling 21 distinct classifications. Specifically, compared with other approaches, the average PSNR value of RHTN at the three magnifications is 1.673 dB higher than Bicubic interpolation, 0.236 dB higher than EDSR-B, 0.210 dB higher than IMDN, 0.210 dB higher than SwinIR-L, 0.268 dB higher than HNCT, 0,271 dB higher than FENet, 0.159 dB higher than Omni-SR, 0.058 dB higher than SRFormer-L, 0.039 dB higher than CAMixerSR. For SSIM, the average performance of RHTN is 0.0432 higher than Bicubic interpolation, 0.0042 higher than EDSR-B, 0.0044 higher than IMDN, 0.0034 higher than SwinIR-L, 0.0042 higher than HNCT, 0,0061 higher than FENet, 0.0029 higher than Omni-SR, 0.0006 higher than SRFormer-L, and 0.0010 higher than CAMixerSR. For LPIPS, RHTN also achieves the best performance, with its average value reduced by 0.0022 and 0.0028 compared to SRFormer-L and CAMixerSR, respectively. Despite being constrained by model capacity, RHTN still demonstrates superior performance on the WHU-RS19 dataset in terms of PSNR, SSIM, and LPIPS metrics compared to competing approaches.
3. Quantitative Results on COWC Dataset: Taking the COWC dataset as an example. As shown in Table 2, the proposed RHTN demonstrates exceptional performance across all metrics on the COWC test dataset. Specifically, compared with other approaches, the average PSNR value of RHTN at the three magnifications is 2.227 dB higher than Bicubic interpolation, 0.212 dB higher than EDSR-B, 0.165 dB higher than IMDN, 0.135 dB higher than SwinIR-L, 0.215 dB higher than HNCT, 0,262 dB higher than FENet, 0.136 dB higher than Omni-SR, 0.043 dB higher than SRFormer-L, 0.034 dB higher than CAMixerSR. For SSIM, the average performance of RHTN is 0.0410 higher than Bicubic interpolation, 0.0048 higher than EDSR-B, 0.0044 higher than IMDN, 0.0037 higher than SwinIR-L, 0.0047 higher than HNCT, 0,0060 higher than FENet, 0.0034 higher than Omni-SR, 0.0005 higher than SRFormer-L, and 0.0006 higher than CAMixerSR. For LPIPS, although SRFormer-L attains performance levels similar to RHTN, the latter exhibits markedly better parameter efficiency.

To delve deeper into the reasons behind the observed phenomenon, this study conduct a comprehensive discussion on the quantitative performance of different methods across various classes within the same dataset. For intuitive analysis of experimental results, this study select five best-performing methods for comparison with RHTN. The per-class PSNR evaluation at a $\times$4 up-scaling factor for the RSSCN7 and WHU-RS19 datasets are presented in Tables 8 and 9, respectively. For the RSSCN7 dataset, RHTN demonstrates strong performance in various land cover types, achieving the highest in six out of seven classes: Grass (33.704 dB), Field (32.933 dB), Industry (26.089 dB), Forest (28.118 dB), Resident (25.073 dB), and Parking (25.323 dB). In the remaining class–River Lake (30.840 dB, second)–although it does not achieve the highest score, the RHTN delivers highly competitive results, coming very close to the best performance. The model achieves the highest average PSNR of 28.869 dB across all classes.

**Table 8:** Mean PSNR (dB) of each class for upscaling factor $\times$4 on the RSSCN7 test dataset. Bold indicates the optimal performance, and underlining indicates the second-best performance.

Class Name	IMDN	SwinIR-L	Omni-SR	SRFormer-L	CAMixerSR	RHTN
Grass	33.663	33.699	33.690	33.689	33.690	**33.704**
Field	32.860	32.894	32.889	32.919	32.915	**32.933**
Industry	25.890	25.938	25.936	26.012	26.028	**26.089**
River Lake	30.822	**30.849**	30.834	30.818	30.810	30.840
Forest	28.060	28.077	28.087	28.105	28.107	**28.118**
Resident	24.907	24.941	24.947	25.019	25.022	**25.073**
Parking	25.153	25.215	25.191	25.284	25.281	**25.323**
Avg	28.765	28.802	28.796	28.835	28.836	**28.869**

**Table 9:** Mean PSNR (dB) of each class for upscaling factor $\times$4 on the WHU-RS19 test dataset. Bold indicates the optimal performance, and underlining indicates the second-best performance.

Class Name	IMDN	SwinIR-L	Omni-SR	SRFormer-L	CAMixerSR	RHTN
Airport	28.410	28.461	28.469	28.531	28.535	**28.588**
Beach	44.889	44.600	44.663	45.000	45.189	**45.234**
Bridge	34.537	34.678	34.664	34.808	34.821	**34.843**
Commercial	25.334	25.345	25.352	25.415	25.427	**25.483**
Desert	39.508	39.625	39.569	39.707	39.714	**39.728**
Farmland	36.418	36.450	36.485	36.555	36.579	**36.633**
Football Field	29.240	29.298	29.280	29.374	29.411	**29.458**
Forest	28.314	28.342	28.369	28.385	28.394	**28.415**
Industrial	27.667	27.686	27.700	27.766	27.797	**27.882**
Meadow	36.601	36.619	36.623	36.654	36.655	**36.666**
Mountain	25.203	25.238	25.238	25.265	25.259	**25.284**
Park	28.463	28.508	28.479	28.501	28.513	**28.558**
Parking	28.250	28.187	28.484	28.631	28.678	**28.941**
Pond	32.387	**32.413**	32.398	32.322	32.324	32.364
Port	27.898	27.931	27.968	28.064	28.090	**28.201**
Railway Station	26.933	27.021	27.015	27.165	27.127	**27.213**
Residential	25.663	25.612	25.714	25.750	25.788	**25.891**
River	28.961	28.985	28.988	29.026	29.020	**29.044**
Viaduct	26.709	26.759	26.783	26.862	26.858	**26.933**
Avg	30.599	30.619	30.644	30.725	30.746	**30.808**

For the WHU-RS19 dataset, RHTN demonstrates consistent superiority across diverse scene types, achieving the highest PSNR in 18 out of 19 classes: Airport (28.588 dB), Beach (45.234 dB), Bridge (34.843 dB), Commercial (25.483 dB), Desert (39.728 dB), Farmland (36.633 dB), Football Field (29.458 dB), Forest (28.415 dB), Industrial (27.882 dB), Meadow (36.666 dB), Mountain (25.284 dB), Park (28.558 dB), Parking (28.941 dB), Port (28.201 dB), Railway Station (27.213 dB), Residential (25.891 dB), River (29.044 dB), and Viaduct (26.933 dB). The experimental results indicate that the proposed RHTN demonstrates superior performance in scenes with intricate textural details, such as airports, commercial districts, parking lots, and sparsely populated residential areas, achieving the highest PSNR values in most cases. Conversely, satisfactory PSNR results are typically observed in more uniform, low-texture environments, such as pond and river lake. These latter scenes lack sufficient discriminative features for effective reconstruction, as the proposed RHTN primarily enhances high-frequency details to improve resolution. In such cases, the scarcity of structural information may limit reconstruction fidelity. Furthermore, the PSNR metric’s suitability for evaluating detail enhancement in complex scenes is notable, whereas its effectiveness may diminish in low-texture regions where perceptual quality improvements are not fully captured by this conventional metric.

**Fig. 8:**
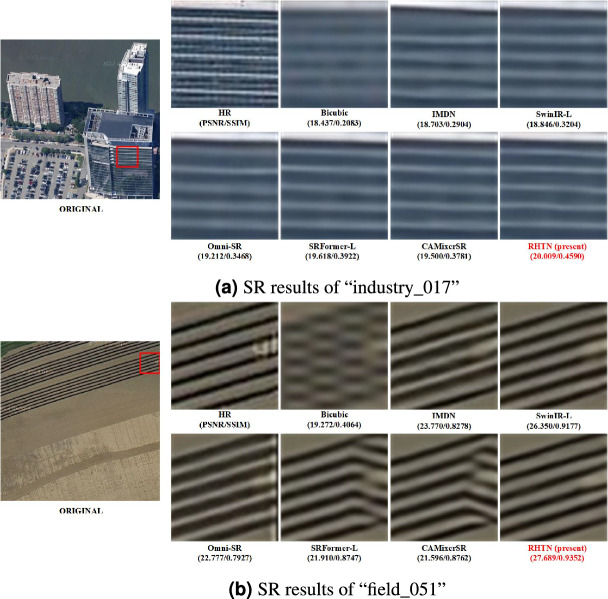
Visual comparison of reconstruction results using samples on RSSCN7 dataset for $\times$4 SR.

**Fig. 9:**
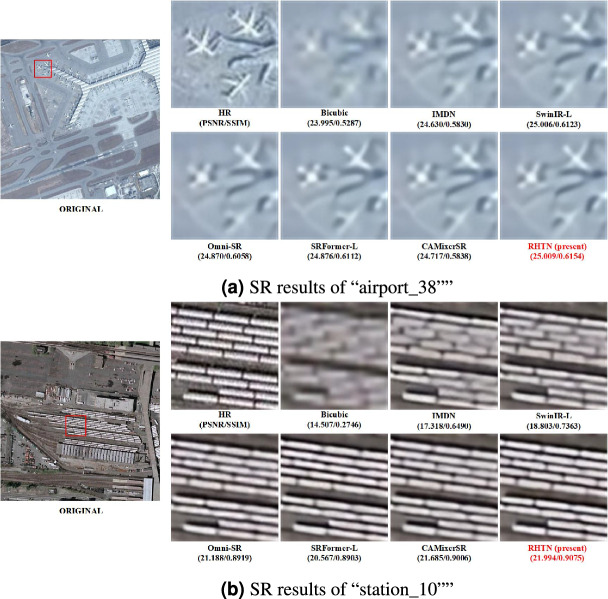
Visual comparison of reconstruction results using samples on WHU-RS19 dataset for $\times$4 SR.

**Fig. 10:**
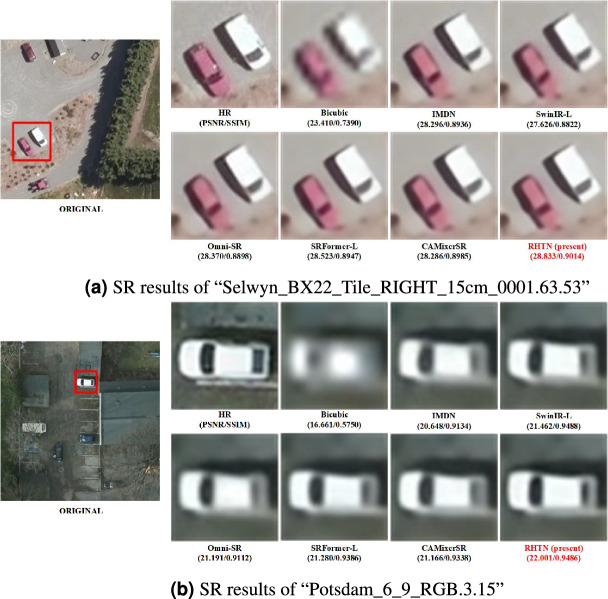
Visual comparison of reconstruction results using samples on COWC dataset for $\times$4 SR.

#### *Visual comparisons*

In addition to the quantitative comparisons discussed above, this study provides a qualitative comparison of the recovered results with different methods. Fig. 8 presents the visual results for tow scenarios from the RSSCN7 dataset: industry and field. In the case of “industry_017”, the building reconstructed by the Bicubic is quite blurry. Compared to IMDN, SwinIR-L, HNCT, Omni-SR, and CAMixerSR, RHTN achieves the highest structural integrity while exhibiting minimal distortion. For “field_051”, the lines recovered by Bicubic have a mosaic effect and become serrated. The CAMixerSR has the second-worst result, attribute to significant spatial distortion. More recent methods (IMDN, SwinIR-L, HNCT, and Omni-SR) can obtain global contrast information, but with significant unreal artifacts. In comparison to other approaches, RHTN achieves clearer edges and produces a sharper image.

Figure 9 shows two examples of the WHU-RS19 dataset. For “airport_38”, we observe that RHTN produces results that are visually close to the ground truth, while other competitive Transformer-based models without the hierarchical windows, such as SwinIR-L, HNCT, Omni-SR, and CAMixerSR, struggle to restore severely degraded details. In the case of “station_10”, we observe that the RHTN obtains more promising results with fewer jaggies and ringing artifacts, and meanwhile reconstructs clearer image contours than the compared advanced approaches. These visualizations demonstrate that our model successfully recovers feature information with rich high-frequency details in remote sensing scenes, yielding superior SR outputs.

Figure 10 shows two examples of the COWC dataset. Compared to other methods, the RHTN demonstrates uperior performance in preserving color fidelity, texture integrity, and edge sharpness of vehicle targets while maintaining improved structural alignment with ground-truth annotations.

## Conclusions

This work presents a novel model named RHTN, a Transformer-based SR reconstruction approach for remote sensing images. The core innovation of our approach lies in the RHTB, which serves as the foundational building block of RHTN. Within each RHTB, expanding hierarchical window mechanisms‌ are implemented to facilitate long-range dependency modeling‌ and harness multi-scale feature representations‌, thereby enhancing super-resolution performance‌. To address the quadratic computational complexity inherent in conventional W-SA mechanism, we design a novel S-CSAHW that enables the network to efficiently capture both spatial structural information and channel-wise features through a hierarchical window framework while achieving linear computational complexity relative to window dimensions. Furthermore, we systematically augment the model’s representational capacity by incorporating the SGFN, which provides additional non-linear spatial modeling capabilities with fewer parameters. Extensive experimental validation demonstrates that RHTN over state-of-the-art methods on both quantitative metrics and visual quality assessments. In future work, we plan to explore the use of RHTN in other remote sensing applications, such as land-cover classification, object detection, and image fusion.

While the RHTN demonstrates superior performance in remote sensing image SR, it inherits inherent limitations common to supervised learning frameworks, it inherits inherent limitations common to supervised learning frameworks. The model’s training paradigm necessitates extensive paired datasets consisting of HR ground-truth images and their degraded counterparts. However, acquiring such precisely aligned image pairs with consistent degradation characteristics remains particularly challenging in remote sensing applications. These constraints motivate our focus on developing semi-supervised or unsupervised learning strategies in subsequent work, which could significantly reduce dependency on paired datasets while maintaining reconstruction quality. For example, by leveraging the intrinsic statistical properties of LR images or modeling the image degradation process based on remote sensing imaging mechanisms, we can reduce dependence on paired training data, thereby better aligning with the requirements of real-world applications.

## Data Availability

The datasets used and/or analyzed during the current study are available from the corresponding author upon reasonable request.
